# Editorial for the Special Issue: Geriatric Nursing Nutrition

**DOI:** 10.3390/nu16244420

**Published:** 2024-12-23

**Authors:** Edda Cava, Mauro Lombardo

**Affiliations:** 1 Clinical Nutrition and Dietetics, San Camillo Forlanini Hospital, Rome Cir.ne Gianicolense 87, 00152 Roma, Italy; 2 Department for the Promotion of Human Science and Quality of Life, San Raffaele Open University, Via di Val Cannuta, 247, 00166 Rome, Italy; mauro.lombardo@uniroma5.it

Malnutrition, a significant risk factor for mortality and morbidity in the elderly, poses a huge threat in the geriatric population, showing a high prevalence, especially in people affected by chronic non-communicable age-related diseases. Moreover, the rapid trend in growth of the elderly populations worldwide highlights the urgency of addressing nutritional management and personalized optimal nutrition to enhance the well-being of the elderly, healthy aging for the population, and at the same time, the prevention and treatment of conditions of geriatric syndromes such as frailty, sarcopenia, osteosarcopenia, or obesity and metabolic diseases related to malnutrition for excess.

The obesity pandemic is a problem that affects any age, from the pediatric to the adult and geriatric populations. Attention should be paid not only to people living independently but also to those living in nursing home facilities and hospital. This is confirmed by the high prevalence found by Bauer et al. in a total of 1236 nursing homes where 16.7% of patients were obese. They described an OR of 2.111 for urinary incontinence in the presence of obesity, but other care problems related to the loss of muscle mass and function should also represent a concern, requiring special attention to be paid to patients’ nutritional status [1]. Moreover, a common condition among the elderly is the combination of loss of muscle mass with the presence of excessive adipose tissue, the osteosarcopenic obesity that aggravates related metabolic issues.

On the contrary, adherence to a Mediterranean Diet is inversely associated with the severity of symptoms related to anxiety and stress but not depression [2]. These findings from Allcock et al. suggest that special attention should be paid to the quality of diet, an important modifiable risk factor for mental health disorders, which could improve the psychological well-being and quality of life in the elderly.

Furthermore, the prevention of cognitive decline through a balanced diet and specific nutritional interventions emerges as an area of growing interest. The role of nutrient intake in preserving lean mass and muscle function is shown by Borda et al., suggesting that early interventions could slow the physical decline associated with old age [3].

Personalized nutrition should be designed for each patient, especially for older people to ensure their compliance to dietary prescriptions required to cover their nutritional and hydration needs. Indeed, in a Chilean group of older people included in the government “Program for Complementary Food in Older People” (PACAM), in which free instant foods fortified with micronutrients were provided, leucine intake was found to be inadequate, and high prevalence rates of sarcopenia were found in the cluster studied by Bustos-Arriagada et al. [4].

Technology represents a mode of support for the management of complex clinical situations when maintaining an adequate nutritional status is pivotal for healthy aging. To give an example, Keller et al. tested a multicomponent intervention including an educational video to improve knowledge and confidence to support staff in order to increase resident fluid intake between meals, avoiding common complications in the care facilities like falls and hospitalization for dehydration [5]. At the same time, technology could support the management of diabetes mellitus in the elderly by a mobile application for monitoring glycated hemoglobin in elderly patients with type 2 diabetes mellitus. Trombini et al. demonstrated how this support led to significant improvements in glycemic control while reducing complications [6].

In the end, the quality of “end-of-life” care is essential and should be included in the multimodal care of an older population growing in both age and number. With this aim, Alford et al. explored the complex and delicate issue of end-of-life nutrition care decisions for healthcare providers and families of residents living in long-term care settings [7].

While guidelines are clear and available on clinical nutrition and hydration in geriatrics, but also in the care of individuals with dementia and palliative care [8,9], multiple situations require prior standardization, but then, a multidisciplinary approach and a long-term follow up is required to improve quality of life in the elderly. To give an example, efforts have been made to address frequent problems in the elderly, such as dysphagia, and standardized terminology and definitions for texture-modified foods and liquids have been provided for all ages [10]. Unfortunately, such a common situation in elderly people can be aggravated by many different diseases, limiting food and liquid intake; therefore, tailored treatment and solutions should be designed for each patient. 

In conclusion, to improve quality of life and healthcare in an aging society, we recently reviewed the fundamental role of nutrition and many of its aspects, focusing on promoting healthy aging and managing problems such as malnutrition and overeating [11]. Nutritional support should be personalized with long-term follow ups to cure or prevent geriatric syndromes such as frailty, sarcopenia, osteosarcopenia, obesity, and metabolic syndrome. Moreover, unique situations that are frequent in the elderly, such as dysphagia, which can reduce nutrient and fluid intake, require special attention to prevent malnutrition and its complications and to maintain muscle mass and bone health in old age.

Overall, the articles collected in this Special Issue provide a comprehensive overview of targeted nutritional strategies for the geriatric population. These papers emphasize the importance of personalized dietary approaches that take into account specific age-related physiological and metabolic needs and demonstrate how nutrition can have a positive and significant impact on the health and well-being of the elderly.
